# Burden of suicide in Poland in 2012: how could it be measured and how big is it?

**DOI:** 10.1007/s10198-017-0892-8

**Published:** 2017-04-08

**Authors:** Katarzyna Orlewska, Ewa Orlewska

**Affiliations:** 1 Medical University of Warsaw, ul. Zwirki i Wigury 61, 02-091 Warsaw, Poland; 2 Faculty of Medicine and Health Sciences, The Jan Kochanowski University in Kielce, AL. IX Wiekow Kielc 19, 25-317 Kielce, Poland

**Keywords:** Years of expected life lost, Premature mortality costs, Suicide, Poland, Burden of disease, I18

## Abstract

**Objectives:**

The aim of our study was to estimate the health-related and economic burden of suicide in Poland in 2012 and to demonstrate the effects of using different assumptions on the disease burden estimation.

**Methods:**

Years of life lost (YLL) were calculated by multiplying the number of deaths by the remaining life expectancy. Local expected YLL (LEYLL) and standard expected YLL (SEYLL) were computed using Polish life expectancy tables and WHO standards, respectively. In the base case analysis LEYLL and SEYLL were computed with 3.5 and 0% discount rates, respectively, and no age-weighting. Premature mortality costs were calculated using a human capital approach, with discounting at 5%, and are reported in Polish zloty (PLN) (1 euro = 4.3 PLN). The impact of applying different assumptions on base-case estimates was tested in sensitivity analyses.

**Results:**

The total LEYLLs and SEYLLs due to suicide were 109,338 and 279,425, respectively, with 88% attributable to male deaths. The cost of male premature mortality (2,808,854,532 PLN) was substantially higher than for females (177,852,804 PLN). Discounting and age-weighting have a large effect on the base case estimates of LEYLLs. The greatest impact on the estimates of suicide-related premature mortality costs was due to the value of the discount rate.

**Conclusions:**

Our findings provide quantitative evidence on the burden of suicide. In our opinion each of the demonstrated methods brings something valuable to the evaluation of the impact of suicide on a given population, but LEYLLs and premature mortality costs estimated according to national guidelines have the potential to be useful for local public health policymakers.

## Introduction

Suicide accounted for 1.4 and 1.48% of all deaths worldwide in 2012 and 2015, respectively [1, 2], making it the 14th leading cause of death [2]. In Poland it was the leading cause of death among people aged 15–39 [3]. The World Health Organisation (WHO) recognizes suicide as a public health priority, calls for action to address this problem and encourages countries to develop or strengthen comprehensive suicide prevention strategies [1]. In debates on research funding and public health issues it is necessary to quantify the burden of suicide, both health-related and economic. A variety of different metrics is available to estimate the health-related impact any given event or disease has on society: e.g., number of deaths, mortality rate (crude or standardized), and years of life lost (YLL). Traditional mortality statistics (number of deaths, mortality rate) deny the fact that death at a young age is, compared with death at an advanced age, generally considered to be a greater loss not only to an individual, but to the society as well. YLL, however, weighs deaths at a young age more heavily than those at a more advanced age [4–6]. Besides the obvious advantages of using the YLL, one may encounter difficult issues, e.g., theoretical and philosophical problems of discounting the value of life lived in the far future, age-weighting and the practical problem of using life tables (either standard reference or country-specific life tables). Although the GBD (global burden of disease) Mortality and Causes of Death Collaborators, a large international consortium of researchers led by the Institute for Health Metrics and Evaluation (IHME) attempted to standardize YLL with respect to standard model life tables [2, 7, 8], authors of recently published studies still use alternative standards [9–13]. Manipulations of YLL—discounting, age-weighting, age-standardizing—are also variously applied [9–13]. This methodological diversity results in the fact that YLL is not routinely used by policymakers for measuring and monitoring the impact of local efforts to reduce premature mortality in a given population. From the economic point of view, every suicide-related death of someone of working age represents a financial loss to society. One of the ways to measure the economic impact of suicide is to estimate the cost of lost productivity due to suicide-related premature mortality. The aim of the study is to apply a consistent methodology for population-based data to estimate the health-related and economic impact of suicide in Poland. We have examined the effect of replacing standard model life tables with Polish life-expectancy values to illustrate how each method of calculating YLL provides a different value of the burden suicide has on that society. We have used different scenarios in our YLL calculations to demonstrate the effects of time-discounting and age-weighting on the disease burden estimation. To allow the interpretation of results, we referred suicide-related estimates to the overall mortality burden of all causes of death in Poland in 2012.

## Methods

Absolute numbers of suicide-related deaths and all causes of death by sex and 5-year age groups (0–4, 5–9, 10–14, (…), 75–79, 80–84, >85) were extracted from the Polish Central Statistics Office database. We have focused on the most recent complete data, which was for the year 2012. YLL was calculated as country-specific (local) expected years of life lost (LEYLL) and standard expected years of life lost (SEYLL). Country-specific life expectancy values for each 5-year age group for males and females living in Poland was derived from life tables for 2012, with a life expectancy of 80.98 years at birth in women and 77.71 years in men [14]. Each LEYLL value was calculated by multiplying the mortality values by the remaining life expectancy values for each age category and than summed to illustrate the overall LEYLL. No cutoff for age was used for the calculation, age at death at each interval was a midpoint of the range, e.g., each death in the 25–29 age group was considered to be 27.5. In the base case analysis LEYLL was computed with a time discount rate at 3.5% and no age-weighting, as recommended by the Polish health technology assessment guidelines [15]. Mean LEYLL was measured by dividing the overall LEYLL by the number of deaths. SEYLL was determined by the average life expectancy at the age of death, using the normative survivorship derived from a model life table. In the base case calculation we have used the most recent WHO Global Health Estimates (WHO GHE) standard life table, which is based on the frontier national life expectancy projected for the year 2050 by the World Population Prospects 2012 and gives a life expectancy of 91.9 years at birth for both sexes [16]. The SEYLL was calculated with a time discount rate at 0% and no age-weighting, as was recommended by GBD and adopted by WHO [2, 7, 16].

Costs of premature mortality were estimated using the human capital approach, which measures lost productivity with regard to the forgone earnings [17]. For each death over a working lifetime (>15 years and < the retirement age, which in Poland is 60 years for women and 65 for men), years of potential productive life lost (YPPLL) were calculated and then valued using sex-specific annual wages from the age of death until the retirement age. Costs were adjusted for unemployment and labor force participation rates according to labor force characteristics in 2012 [18], and discounted at 5% per annum [15]. Based on the analysis of economic activity rate by sex in the years 2010–2013 [18], it was assumed that the economic activity rate for males and females will increase annually by 1.3 and 0.5%, respectively. Future wage growth was estimated at 3.4% based on average country-specific GDP growth from 2000 to 2012. Cost estimates were subsequently summed over deaths in each 5-year age group and across age groups to provide total cost of lost productivity due to suicide-related premature mortality separately for female and male populations and for both sexes combined. In addition, premature mortality costs were expressed per single suicide-related death and per 1000 persons. Costs were expressed in Polish zloty (PLN) (1 euro = 4.3 PLN in 2016).

Deterministic sensitivity analysis was performed to assess the impact of changes in key parameters on base-case estimates of LEYLL, SEYLL and premature mortality costs. Given that the original GBD 1990 study and subsequent WHO updates have applied discounting and age-weighting to compute YLL, a 3% discount rate and standard age-weights rate, which gives less weight to years of healthy life lost at young ages and older ages, were used in our calculation of LEYLL [19, 20]. In addition, a 0% discount rate was applied in the LEYLL calculation to account for more recent WHO and GBD recommendations [2, 7, 16] and Polish health technology assessment guidelines for sensitivity analyses [15].

In the sensitivity analysis, we calculated SEYLL by applying the reference life tables used previously in the GBD studies: (1) West Level-26 with a life expectancy of 80 years at birth for males and 82.5 years for females, age-weighted and discounted, (2) West Level-26 with a life expectancy of 80 years at birth for males and 82.5 years for females, not age-weighted nor discounted, and (3) GBD 2010 with a life expectancy of 86 years at birth for both males and females, not age-weighted nor discounted [21]. Additionally, we calculated SEYLL applying the up-to-date reference life tables proposed by IHME and recently used in GBD 2015, with a normative standard life expectancy at birth of 86.59 years, not age-weighted nor discounted [2].

For premature mortality cost, different discount rates (0 and 3.5%) were tested [15]. To take into account the uncertainty over future growth in the Polish economy, 0% wage growth and 0% economic activity growth were applied. The estimates of unemployment rates from 2015 were used to reflect more up-to-date changes in the labor market [22]. Moreover, to account for the change in the official retirement age to be implemented in Poland in the near future, the effect of extending the retirement age to 68 for both males and females was explored.

## Results

### Base case analysis

#### Number of deaths, LEYLL and SEYLL overall and by sex

**Fig. 1:**
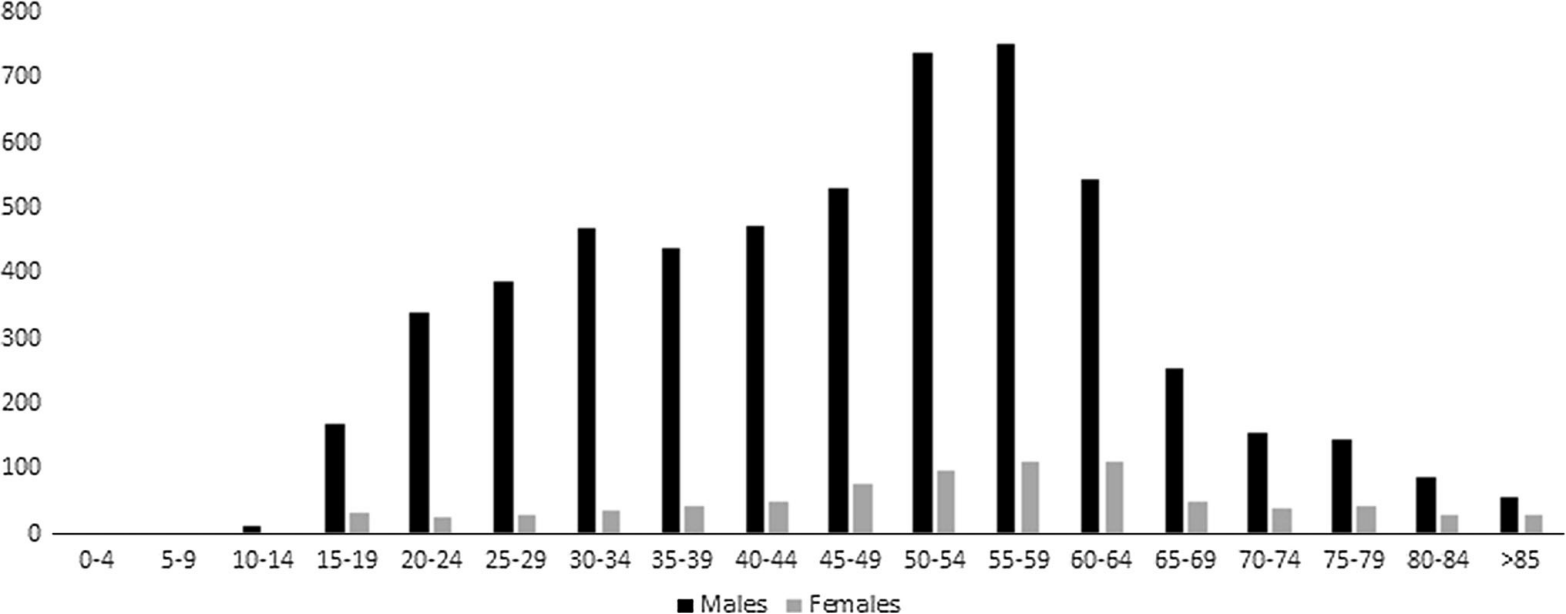
Absolute numbers of suicide-related deaths in Poland in 2012

A total of 6365 suicides were reported in Poland in 2012, 87% of which were among males. The absolute number of deaths in each 5-year age category is presented in Fig. 1. The highest mortality for both sexes was observed among people aged 50–64 (2035 males and 319 females); the average age at death was 48 for males and 54 for females. For comparison, the average age of all-cause mortality was 68.7 for males and 77 for females.

**Fig. 2:**
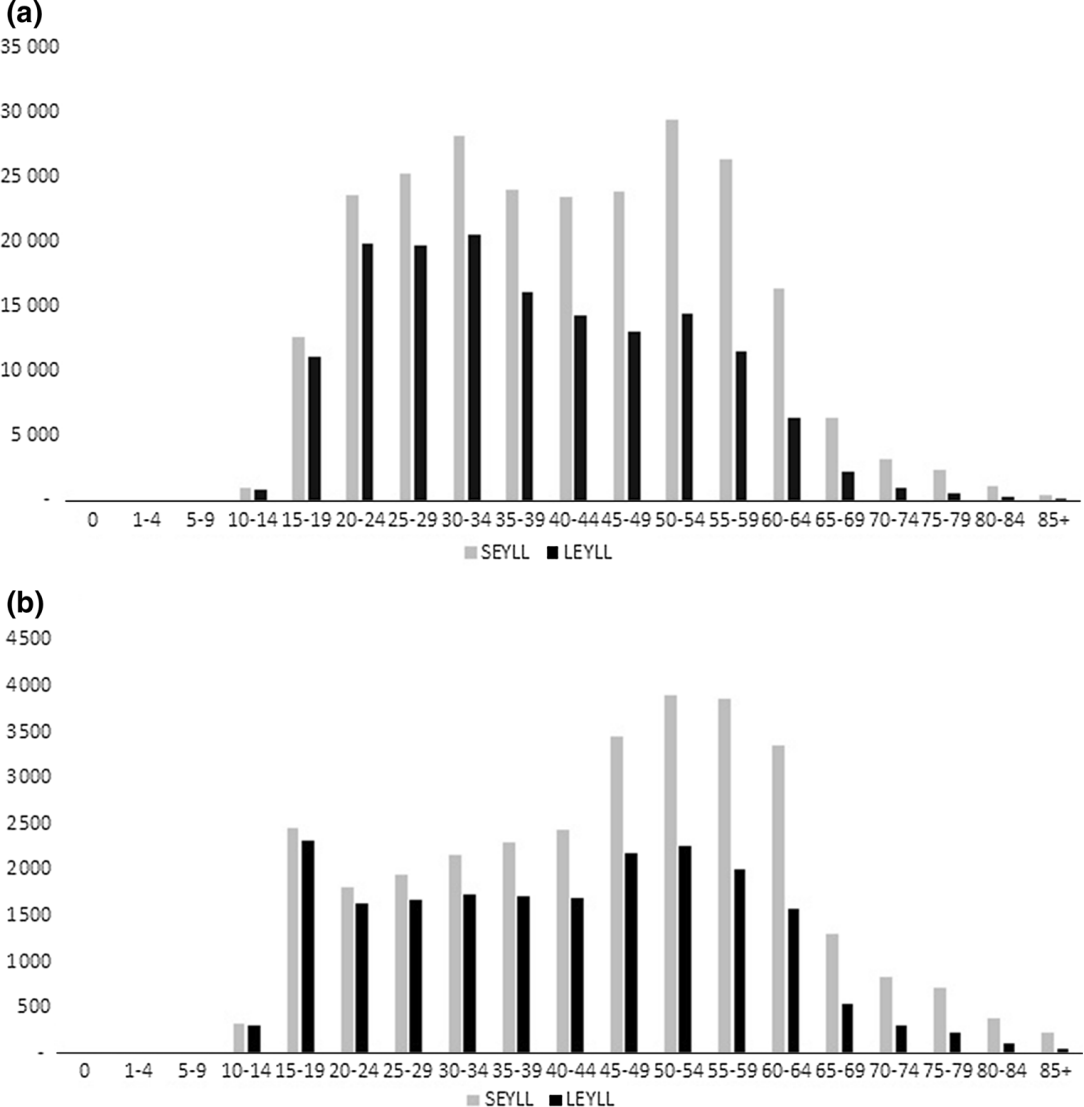
**a** Comparison of the male SEYLL and LEYLL age distribution. **b** Comparison of the female SEYLL and LEYLL age distribution

The total LEYLL amounted to 109,338 and was substantially higher among males than among females (96,388 vs 13,950) (Table 1). Similarly, LEYLL per 1000 persons was 7 times higher among males, compared to females. This disparity was caused by a notable difference in the mortality between sexes: the male mortality in each 5-year age category was many times greater than that of females (Fig. 1). However, because of the higher life expectancies for women, the mean LEYLL was similar for females and males: 17.22 and 17.17, respectively. The total SEYLL attributable to suicide amounted to 279,425, 89% of which was among males. Both mean SEYLL and SEYLL per 1000 persons was higher in males (45 and 13.3, respectively) than in females (39 and 1.6, respectively). A comparison of the SEYLL and LEYLL age distribution (Fig. 2) revealed that the effect of using loss function corresponding to longer life expectancies is more pronounced in the more advanced age groups. The strongest impact of prolonged life-expectancy was identified for women aged 80 and older and for men aged 75 and older, with SEYLL being 4 times higher than LEYLL.

**Table 1:** Sensitivity analyses for LEYLL according to different assumptions for the discount rate and age-weighting

	Males				Females				Both sexes combined			
	LEYLL	% Change from BC	LEYLL per death	LEYLL per 1000	LEYLL	% Change from BC	LEYLL per death	LEYLL per 1000	LEYLL	% Change from BC	LEYLL per death	LEYLL per 1000
Base case (BC)	95,388		17.2	5.1	13,950		17.2	0.7	109,338		17.2	2.9
Discount rate (BC: 3.5%)												
3%	101,841	7%	18.3	5.5	14,936	7%	18.4	0.8	116,777	7%	18.4	3.0
0%	160,965	69%	29	8.6	24,335	74%	30	1.2	185,300	41%	29.1	4.8
Age weights (BC: no age weights)												
Age weights	122,145	28%	21.99	6.5	16,271	17%	20.09	0.8	138,416	27%	21.75	3.59
Age weights (BC: no age weights, 3.5% discount rate)												
Age weights and 0% discount rate	152,404	59%	27.44	8.2	20,370	46%	25.15	1.0	172,774	58%	27.4	4.48

**Fig. 3:**
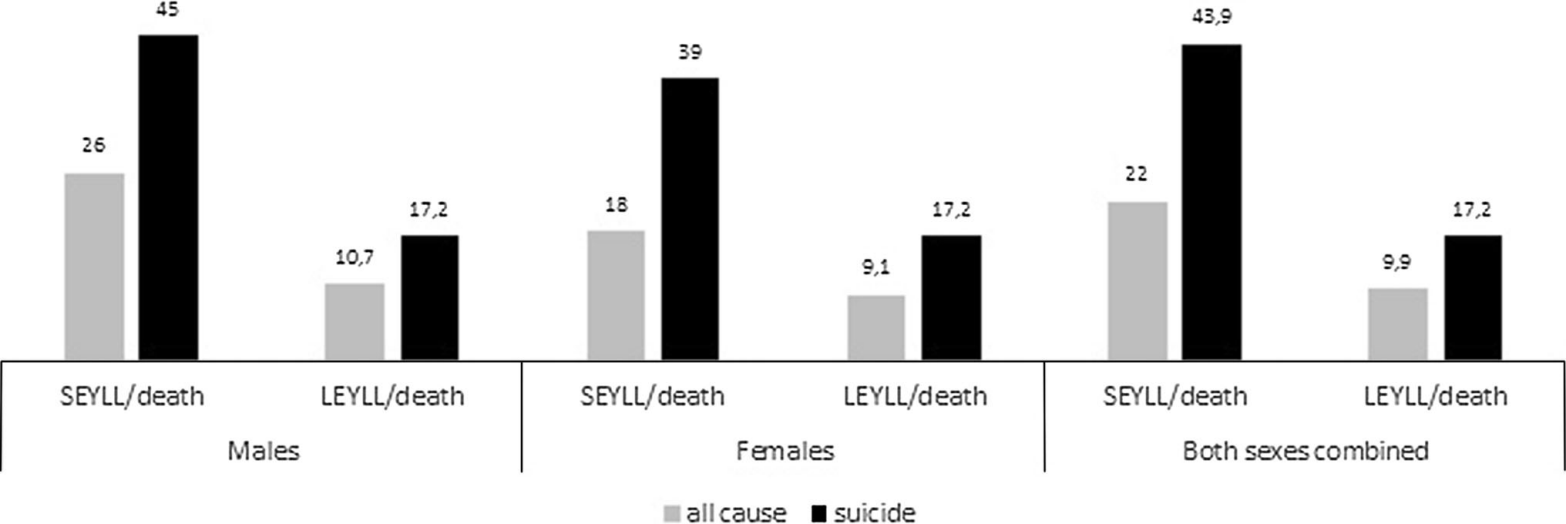
Comparison between mean suicide-related LEYLL and SEYLL and mean all-cause death-related LEYLL and SEYLL in the Polish population

The LEYLL and SEYLL due to all-cause mortality for both sexes in Poland were 3,817,452 and 8,457,390, respectively. When these values were taken as point of reference, the loss of life years due to suicide accounted for 3% of all-cause mortality-related LEYLL and 3% of all-cause mortality-related SEYLL. Both mean suicide-related LEYLL and SEYLL exceeded mean all-cause mortality-related LEYLL and SEYLL, and the excess was greater for females than males (Fig. 3).

#### Premature mortality costs overall and by sex

In the base case analysis, the total cost of lost productivity due to suicide-related premature mortality was 2,986,707,338 PLN, 94% of which was among males (2,808,854,532 PLN). The male cost of lost productivity per premature suicide death exceeded the female cost by 130% (505,644 PLN vs 219,571 PLN). The cost per 1000 persons was 150,614 PLN and 11.04 PLN for males and females, respectively.

The all-cause premature mortality cost was 30,991,739,990 PLN (26,786,245,030 PLN for males and 4,205,494,960 PLN for females), which represents 804,285 PLN per 1000 persons (1,436,311 per 1000 males and 211,502 PLN per 1000 females). Mean cost of lost productivity was 80,542 PLN (132,543 PLN per dead male and 23,013 per dead female). In both sexes combined and among males, suicide contributed to 10% of overall premature mortality costs, while female suicide-related premature mortality cost represented 4% of the overall cost. For both males and females, the cost of premature mortality per suicide-related death exceeded that of all causes of death. The excess was more pronounced in the female population (219,571 PLN vs 23,013) than in the male population (505,644 PLN vs 132,543 PLN).

### Sensitivity analysis

Discounting and age-weighting have a large effect on the base case estimates of LEYLL. With a 3% discount rate the total and sex specific LEYLL increases by 7%, with 0% discount rate LEYLL is in comparison to base case by 41% higher for both sexes combined and by 69% and 74% higher for males and females, respectively (Table 1). Applying age weights had greater impact on LEYLL in males (increase by 28%) than in females (increase by 17%). In the 0% discount rate and age-weights scenario, LEYLL was 59% higher in males, 46% higher in females and 58% higher in both sexes combined. By applying the loss function which corresponds to longest life expectancies, SEYLL resulted in an approximately 2.5 times bigger of the total and mean value in comparison to the West Level-26 with age-weighting and discounting (Table 2).

**Table 2:** Sensitivity analyses for SEYLL according to different assumptions for the reference life tables

Reference life tables	Males			Females			Both sexes		
	SEYLL	SEYLL per death	SEYLL per 1000	SEYLL	SEYLL per death	SEYLL per 1000	SEYLL	SEYLL per death	SEYLL per 1000
West Level-26 age-weighted and discounted	104,875	18.9	5.6	13,103	16.2	0.7	117,978	18.54	3.06
West Level-26 not age-weighted and discounted	186,709	33.6	10.0	25,093	30.1	1.3	211,802	33.3	5
GBD 2010	206,152	37.1	11.1	25,610	31.6	1.3	231,762	36.4	6
GBD 2015	222,002	40	11.9	27,808	34	1.4	249,810	39.25	6.5
WHO GHE (base case analysis)	247,928	45	13.3	31,497	39	1.6	279,425	43.9	7

Table 3 presents the results of sensitivity analysis for premature mortality costs. The greatest impact on the estimates of suicide-related premature mortality costs had the value of the discount rate. With a 0% discount rate the total cost was 117% higher in both sexes and in males, and 107% higher in females. An assumption of fixed wage resulted in a 31% lower cost in both sexes and males, and a 30% lower cost in females. Extending the retirement age to 68 for both sexes resulted in an increase of costs by 66% in females, by 15% in males and by 18% in both sexes combined. Varying parameters that characterized the labor market (unemployment rate, economic activity) had a greater impact on costs in the female population than on costs in the male population.

**Table 3:** Sensitivity analyses for premature mortality costs according to different assumptions for the discount rate, wage growth, labor market characteristics and the retirement age

	Males				Females				Both sexes combined			
	Total premature mortality cost (PLN)	% Change from BC	Premature mortality cost per death (PLN)	Premature mortality cost per 100,000 (PLN)	Total premature mortality cost (PLN)	% Change from BC	Premature mortality cost per death (PLN)	Premature mortality cost per 100,000 (PLN)	Total premature mortality cost (PLN)	% Change from BC	Premature mortality cost per death (PLN)	Premature mortality cost per 100,000 (PLN)
Base case (BC)	2,808,854,532		505,644	150,614,201	177,852,804		219,571	894,453	2,986,707,338		469,239	7,750,978
Discount rate (BC: 5%)												
0%	6,107,398,271	117%	1,099,442	32,748,613	367,624,189	107%	453,857	1,848,847	6,475,022,460	117%	1,017,286	16,803,706
3.5%	3,422,131,782	22%	616,045	18,349,887	213,986,854	20%	264,181	1,076,178	3,636,118,636	22%	571,268	9,436,302
Wage growth (BC: 3.4%)												
0%	1,927,450,639	−31%	346,976	10,335,225	124,921,415	−30	154,224	628,252	2,052,272,054	−31%	322,447	5,326,230
Economic activity growth (BC: 0.5% males, 1.3% females)												
0%	2,637,220,280	−6%	474,747	14,141,096	153,618,567	−14%	189,653	772,575	2,790,838,847	−7%	438,466	7,242,668
Unemployment (BC: 2012 rates)												
2015 rates	2,886,628,993	3%	519,645	15,478,456	185,309,571	4%	228,777	931,955	3,071,938,563	3%	482,630	7,972,166
Retirement age (60 females, 65 males)												
68 both sexes	3,226,835,071	15%	580,888	17,302,683	295,784,818	66%	365,166	1487,555	3,522,619,890	18%	553,436	9,141,755

## Discussion

Our findings provide quantitative evidence of the health-related and economic impact of suicide. In absolute and relative terms, suicide represents a significant loss to the Polish economy, which accentuates the importance of investing in effective prevention actions. A national suicide prevention program was developed in 2012 and suggested focusing on establishing an efficient collaboration between emergency services, school staff, clergymen and specialist medical units [23]. A comprehensive training program for primary care physicians, schoolteachers and social workers to recognize affective disorders, suicidal thoughts and behavior is hoped to play a major role in preventing the expected growth in suicide-related mortality rates [23]. It is worth mentioning that the efficacy of the first national preventive programs implemented in the USA and some European countries in the 90s and their efficacy has been proven by a notable decrease of reported suicides (30–50% during a period of 5–15 years). Since 1953, when the Samaritans Organisation set in motion a 24-h suicide helpline, Great Britain has noted a systematic decrease in the number of reported suicides, making it one of Europe’s countries with the lowest suicide rate [23].

Our study revealed substantial sex-specific differences in both LEYLL and SEYLL due to suicide and in premature suicide-related mortality cost. Primarily, this is a consequence of a substantially higher rate of fatal suicide attempts among men. Except for China, where more females than males die due to suicide [24], this is a ubiquitous tendency. On average, the global male to female suicide-related death ratio is 3.5:1. In Poland it amounts to a distressing 7:1. Substantial sex-specific differences in costs of premature mortality reflected sex- and age-related variations in labor force participation and earnings. The use of labor market data to derive costs results in the fact that premature male deaths are “weighted” more heavily than those of women. This is because, on average, men have higher labor force participation rates than women and are paid more.

The fact that suicide accounted for 3% of all-cause mortality-related LEYLL and SEYLL and 10% af the total premature mortality cost in Poland would not be expected a priori based on the number of deaths from this cause (2% of all deaths). The analysis and interpretation of death registry using years of lost life and premature mortality costs provide objective evidence for public health policymakers to inform and guide the setting of local public health priorities. Moreover, mean years of lost life and premature mortality cost per death provide an insight to the burden of suicide and the effect it has on an individual, rather than on the population as a whole, and bring attention to the importance of preventing premature, suicide-related deaths.

In our study we have applied a long-established and widely used methodology for high quality population-based data in a consistent and transparent fashion. What makes our study unique is that we have estimated both the health-related and economic burden of suicide, included LEYLL and SEYLL in the health-related estimations of the burden of suicide and performed an extensive sensitivity analysis to determine how different values of key parameters impact the base case estimates. To our knowledge, such a detailed analysis has never been presented on a national level before. In the recently published studies on the burden of external-cause mortality in the Lodz province [25], and premature mortality in Poland [10], years of life lost were counted and analyzed by the method described in GBD 1990 [20]. In these studies a mortality standard norm (West Level-26) had a life expectancy of 80 years at birth for males and 82.5 years for females, SEYLL was computed with a 3% discount rate and age-weighting. Because there have been substantial revisions to the methods of calculating SEYLL, these estimates fail to meet the new methodological requirements and may be comparable only with one of the scenarios presented in the sensitivity analysis.

Studies of the burden of suicide from other countries [26–29] have used a different approach as well—they have calculated the potential years of lost life (PYLL). This methodology, recommended by the Organisation for Economic Co-operation and Development (OECD), uses life limits, e.g., 75 years [27] or 65 years [29], and does not take into account the years lost due to deaths which occur above this age limit.

In our study, we have calculated both LEYLL and SEYLL. It is difficult to indicate which measure may be more useful for decision makers in estimating the health-related burden of suicide. On the one hand, calculating the years of lost life using country-specific life tables reflects a country-specific disease burden and incorporates strategies used in cost-effectiveness analysis. On the other hand, the projected life expectancy is influenced by preventable deaths which currently occur in a given population. The standard reference life table is intended to represent the potential maximum life span of an individual in good health at a given age. Calculating SEYLL may overestimate the years of lost life compared to LEYLL. Nonetheless, SEYLL has several advantages: (1) the projected life expectancy represents the maximum life span of an individual, and (2) it allows comparisons against the same standard. Taking into consideration that country-specific information is of higher importance for local public health policymakers, LEYLL calculations, which are based on country-specific mortality and country-specific expected survival, seem to be more useful than SEYLL. Moreover, this approach is in line with methodological recommendations for health outcome evaluation in the cost-effectiveness analyses [15]. In this context SEYLL can only serve as an additional measure useful for international comparative studies and global health estimates of the burden of diseases, provided that the methods of calculation comply with the working standards and requirements currently in force. Presently, GBD 2015 methodology proposed by IHME represents the up-to-date strategy [2].

In our study the base case estimations of LEYLL were calculated with discounting at 3.5%, as is recommended for health outcome evaluation by the Polish HTA guidelines [15]. In our opinion the evaluation of the burden of mortality due to a given condition should follow these recommendations in order to avoid using parallel, oftentimes inconsistent methods and to avoid decision-making paradoxes when future costs of health interventions are discounted. Critics of discounting argue that there is no intrinsic reason to value a year of health as less important simply because it is in the future. This argument might be accurate if lost of life years had been defined as quantifying loss of health rather than the social value of loss of heath. LEYLL aims to quantify the social value of the loss of health and in our opinion for this reason it should be discounted, especially when is used to inform and guide the setting of local public health priorities. Age-weighting gives less weight to years of healthy life lost at young and older ages [29]. The standard age-weighting formula is: Cxe^−bx^, where x is the concerned age, and C and b are constants commonly set to 0.1658 and 0.04. Age-weighting is based on the theory of human capital, according to which years lived as a young adult are valued higher than years spent as a young child or older adult, because these are the years of peak productivity. As mentioned above, estimating LEYLL quantifies the social value of loss of health, while society’s interest in productivity is better reflected by estimating the loss of productive life years and costs of premature mortality. Given the lack of consensus on social weighting, we recommend calculating LEYLL under different scenarios, at least: “no discounting, no age-weighting”, “discounting at 3 or 3.5%, no age-weighting”, and “discounting and age-weighting”.

The cost of premature mortality was calculated by multiplying the relevant number of lost work years with a wage rate estimate. It has been argued that actual productivity costs may be strongly influenced by compensation mechanisms adjustments [30]. However, evidence on such mechanisms is scarce and, despite a number of studies, a consensus on the methods used to produce productivity cost estimates has not been reached [31]. Such a lack of agreement is a likely reason for ignoring productivity costs in the economic evaluation of the burden of a disease or event.

## Limitations of the study

We are well aware of the limitations of the present study. The reliability of the analysis of Polish population mortality due to suicide depends on the correct classification of the primary cause of death. Our study is based on the data from the register of deaths, run by the Central Statistical Office. Death by suicide was defined as “intentional self-harm” according to the ICD-10 categories X60–X84. This is defined in a way that includes all suicide deaths, but has somewhat wider logical scope. The accuracy of suicide rates in the official reports may be influenced by wrong classification of the cause of death.

## Conclusions

Our study contributes to understanding of the burden of suicide in Poland. Our results strongly indicate that within the public health care sector, suicide prevention is an issue to which priority should be given. It can be stated that each of the demonstrated methods is valuable for the evaluation of the impact of suicide on a given population: LEYLL quantifies the social value of health loss, SEYLL quantifies the health loss, and premature mortality costs reflect the loss of productivity due to deaths. LEYLL and premature mortality costs estimated according to national guidelines have the most significant potential to be used by local public health policymakers.
